# A Novel and Simple Method for Rapid Generation of Recombinant Porcine Adenoviral Vectors for Transgene Expression

**DOI:** 10.1371/journal.pone.0127958

**Published:** 2015-05-26

**Authors:** Peng Zhang, Enqi Du, Jing Ma, Wenbin Wang, Lu Zhang, Suresh K. Tikoo, Zengqi Yang

**Affiliations:** 1 College of Veterinary Medicine, North-west A&F University, Yangling, Shaanxi, China; 2 VIDO-InterVac, University of Saskatchewan, Saskatoon, Saskatchewan, Canada; 3 Vaccinology & Immunotherapeutics Program, School of Public Health, University of Saskatchewan, Saskatoon, Saskatchewan, Canada; 4 Department of Veterinary Microbiology, Western College of Veterinary Medicine, University of Saskatchewan, Saskatoon, Saskatchewan, Canada; Naval Research Laboratory, UNITED STATES

## Abstract

Many human (different serotypes) and nonhuman adenovirus vectors are being used for gene delivery. However, the current system for isolating recombinant adenoviral vectors is either time-consuming or expensive, especially for the generation of recombinant non-human adenoviral vectors. We herein report a new and simple cloning approach for the rapid generation of a porcine adenovirus (PAdV-3) vector which shows promise for gene transfer to human cells and evasion of human adenovirus type 5 (HAdV-5) immunity. Based on the final cloning plasmid, pFPAV3-CcdB-Cm, and our modified SLiCE strategy (SLiCE cloning and lethal CcdB screening), the process for generating recombinant PAdV-3 plasmids required only one step in 3 days, with a cloning efficiency as high as 620±49.56 clones/ng and zero background (100% accuracy). The recombinant PAdV-3 plasmids could be successfully rescued in porcine retinal pigment epithelium cells (VR1BL), which constitutively express the HAdV-5 E1 and PAdV-3 E1B 55k genes, and the foreign genes were highly expressed at 24 h after transduction into swine testicle (ST) cells. In conclusion, this strategy for generating recombinant PAdV-3 vectors based on our modified SLiCE cloning system was rapid and cost-efficient, which could be used as universal cloning method for modification the other regions of PAdV-3 genome as well as other adenoviral genomes.

## Introduction

Adenovirus-based vectors have been demonstrated to be excellent gene delivery vehicles in mammals [1–4], and certain features of these vectors have made them potential candidates for vaccine development and gene therapy [5]. For example, adenovirus-based vectors can deliver specific genes of interest to a broad spectrum of both actively dividing and post-mitotic quiescent mammalian cells. In addition, the genome of adenovirus vectors has large insert capacity, and the transgene can be expressed at a high level by strong heterologous promoters. These vectors can also be easily grown to high titers in tissue culture (10^9^–10^10^ infectious units per ml, IFU/ml). Moreover, the vector genome rarely integrates into the host chromosome and remains primarily episomal; hence, their use is safe, with the low risk of insertional mutagenesis [6]. Human adenovirus serotype 5 (HAdV-5) vectors are well characterized and currently widely used in gene therapy and vaccine development. However, majority of humans contain circulating antibodies against HAdV-5 capsid proteins (fiber, hexon, penton) [7, 8]. In addition, adenovirus often elicits strong innate inflammatory responses within hours after administration, especially in the present of HAdV-5 immunity [9]. Due to the strong activation of innate immune response and the pre-existing immunity (PEI) to HAdV-5, the gene transfer and duration of transgene expression for gene therapy is limited. Meanwhile, the potential toxicity of HAdV-5 to liver and spleen limited the efficacy of vaccine development based on HAdV-5 as well. To circumvent PEI to HAdV-5, one promising alternative being explored is the development of non-human adenovirus vectors [10,11]. Indeed, the absence of cross-neutralization by sera containing HAdV-5-specific antibodies and circumventing the preexisting immunity in humans have helped to initiate the evaluation of porcine adenovirus serotype 3 (PAdV-3) as a vector for the delivery of vaccine antigens in humans [12]. PAdV-3 is also being evaluated as a vaccine delivery vehicle in animals [13–17]. Although PAdV-3 is being developed as a vaccine delivery vehicle, the efficient evaluation of the PAdV-3 vector system requires the rapid generation of PAdV-3 vectors.

The conventional methods for constructing the PAdV-3 vector system are digestion-ligation based on *in vitro* restriction endonucleases and DNA ligases, *in vivo* homology recombination in *E*. *coli* or a combination of both. However, these methods are still limited by several factors, including the difficulty of selecting restriction endonuclease sites, the low efficiency and difficulty in screening for positive homologous recombination and the requirement of additional time and labor. Thus, the availability of an efficient, robust and universal approach for constructing recombinant porcine adenoviral vector is urgently needed.

SLiCE (Seamless Ligation Cloning Extract) is a novel cloning method that utilizes an easy-to-generate PPY strain cell extract to construct complex recombinant DNA molecules in a single in vitro recombination reaction [18]. As the PPY strain was engineered to constitutively express the λ prophage *redβ* and *gam* genes under the control of the EM7 and Tn5 promoters, respectively, and also the *redα* gene under the control of an arabinose-inducible pBAD promoter (araC-pBAD), the cell extract of PPY strain contains the recombinase of redαβγ which could greatly promoted the *in vitro* homologous recombination with 20–40 bp homology.

Compared with the traditional cloning method, SLiCE cloning has several advantages. (1) SLiCE facilitates seamless cloning without leaving any unwanted sequences at the cloning junctions. (2) SLiCE is a time- and labor-saving strategy that allows the generation of more complex recombinant plasmids using multiple inserts in a single cloning reaction with high efficiency and accuracy. (3) SLiCE is highly cost effective, as the SLiCE extract can be obtained from certain laboratory RecA^-^ bacterial strains, such as DH10B and JM109 [18].

In this study, we report for the first time the successful development of an efficient, robust and universal approach for the construction of recombinant porcine adenoviral vectors in a seamless and precise fashion based on our modified SLiCE cloning method.

## Materials and Methods

### Cell culture

Porcine retinal pigment epithelium cells (VR1BL) [19], which were established and stored in our laboratory, were cultured in minimum essential medium (MEM) supplemented with 10% fetal calf serum (FCS). VR1BL cells constitutively express the HAdV-5 E1 and PAdV-3 E1B 55k genes. Swine testicle (ST) cells obtained from ATCC (CRL-1746) were cultured in MEM supplemented with 5% FCS.

### Bacteria, plasmid, virus and viral DNA

*Escherichia coli* strain PPY was a gift from Dr Yongwei Zhang (Department of Cell Biology, Albert Einstein College of Medicine, Bronx, NY 10461, USA). *E*. *coli* DB3.1, which was used for generating a plasmid with the lethal CcdB gene, was purchased from Invitrogen. MAX Efficiency DH10B chemically competent cells were also purchased from Invitrogen. The plasmid pAd-PL/Dust was a gift from Dr Jun Luo (College of Animal Science and Technology, Northwest A&F University, China) and used as a DNA template for the amplification of the CcdB-Cm expression cassette. The plasmid Ad-Track was purchased from Agilent Technologies, Inc. Viral genomic DNA was extracted from recombinant PAV219-infected monolayers of VR1BL cells according to reported methods [19, 20], and the recombinant PAdV-3 plasmids in this study originated from PAdV-3 strain 6618 (GenBank Accession No. AF083132.1; genome size of 34,094 bp).

### Preparation of SLiCE extract

The SLiCE extract was prepared based on the protocol from Dr Zhang [18]. Briefly, *Escherichia coli* strain PPY was grown at 37°C in 2×YT medium until an OD600 > 5.0. The cells were subsequently incubated for 2 h in 2×YT medium containing 0.2% L-arabinose to express the prophage protein λ Redα. The cells were harvested by centrifugation at 5000 g for 20 min at 4°C, washed once with ddH_2_O and resuspended in CelLytic B Cell Lysis Reagent (Sigma). The resuspended cells were incubated at room temperature for 10 min to allow lysis to occur. The cell lysates were then centrifuged at 20,000 g for 2 min at room temperature to pellet the insoluble material, and the resulting supernatants were carefully removed from the cell debris and mixed with an equal volume of 100% glycerol. The mixture was aliquoted into 40–60 μl portions and stored at -80°C.

### Plasmid construction

#### Generation of recombinant plasmid pFPAV3-CcdB-Cm

**Fig 1:**
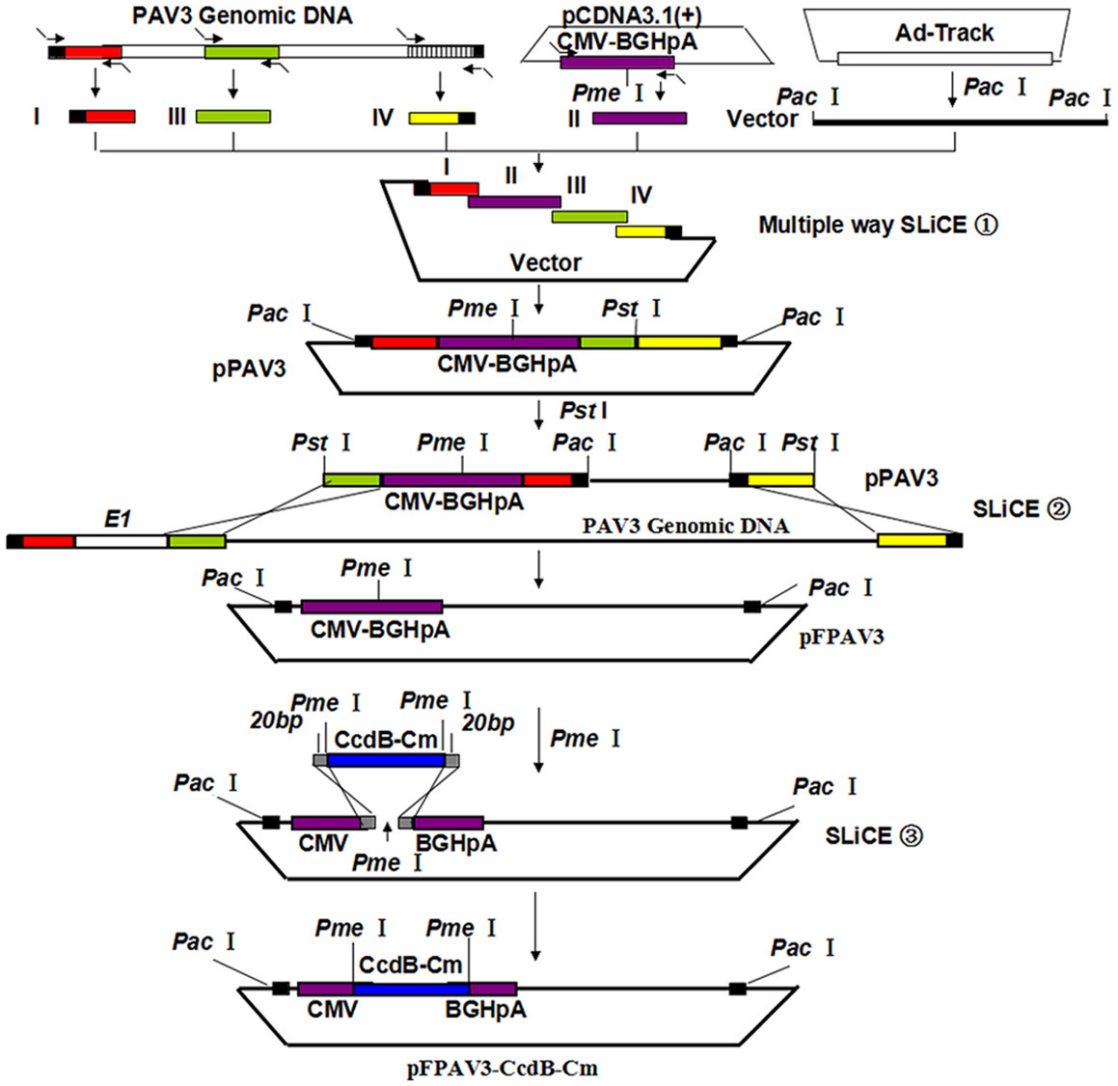
Construction of the template PAdV-3 genome vector for foreign gene insertion. The shuttle plasmid pPAV3 was composed of five fragments: three fragments (1–525 bp, 3273–3678 bp, and 33848–34094 bp) from the wild-type PAdV-3 genome, the fragment of the CMV-*Pme*I-BGHpA cassette from plasmid pCDNA3.1(+) and the fragment of the *Pac*I-linearized pAd-Track vector backbone. The five fragments were flanked with 15-bp homologies in head to tail fashion and assembled in one cloning step to generate shuttle vector pPAV3 based on the multiple-way SLiCE strategy. The transitional genome plasmid pFPAV3 was generated by recombination between *Pst*I-linearized pPAV3 and linear genomic DNA extracted from PAV219 virus-infected VR1BL cells according to a second-round SLiCE strategy. The final genome template of the adenoviral vector pFPAV3-CcdB-Cm was generated by recombination between the *Pme*I-linearized pFPAV3 vector and a PCR fragment of the CcdB-Cm expression cassette flanked with two *Pme*I enzyme sites with 20-bp homologies based on a third-round SLiCE strategy.

A flowchart for construction of the porcine adenoviral vector system is described in Fig 1. Briefly, for the generation of a plasmid harboring the E1-deleted PAdV-3 genome, we first constructed shuttle vector pPAV3, which is composed of five fragments. Each fragment is designed to contain 15–20 bp homologies in head to tail format for assembly in a single cloning step to generate shuttle vector pPAV3 using the multiple-way SLiCE cloning protocol. Three fragments were amplified by PCR using PAdV-3 genomic DNA as the template with the indicated primers: fragmentI(left ITR to 525 bp of the PAdV-3 genome; primers P1 and p2 in Table 1), fragment III (3,273–3,678 bp of the PAdV-3 genome containing pIX; IX Table 1) and fragment IV (33,848–IV genome containing the right ITR and partial E4 region; primers P7 and P8 in Table 1). FragmentII, containing the CMV-*Pme*I-BGHpA cassette, was amplified by PCR using primers P3 and P4 (Table 1) and template pCDNA3.1 (+) DNA. Fragment V, containing a 3-kb *Pac*Ifragment harboring the vector backbone, was isolated by *Pac*Idigestion of the plasmid pAd-track (shuttle vector of Adeasy system). Fragments I, II, III and IV were treated with *Dpn*I prior to purification to remove residual plasmid template DNA. The vector (Fragment V) and these PCR inserts were subjected to gel electrophoresis and purified using the QIAEX II gel extraction kit. The SLiCE reaction mixture contained the following ingredients: 1 μl 10×SLiCE buffer (500 mM pH 7.5 Tris-HCl, 100 mM MgCl_2_, 10 mM ATP, 10 mM DTT), 1 μl linear vector (50 ng), 1 μl insert DNA (containing four inserts with a 1:10 molar ratio of vector to insert), 1 μl SLiCE extract and ddH_2_O in a total volume of 10 μl. The SLiCE reaction mixture was incubated at 37°C for 1 h, and subsequently 1 μl of the SLiCE reaction was transformed into 100 μl MAX Efficiency DH10B chemically competent cells (Invitrogen) following the manufacturer’s instructions. The transformed cells were plated on agar plates containing the appropriate antibiotics.

**Table 1:** Primer and oligonucleotide list.

Fragment		Sequence (5’-3’)
PAV3 genome (1nt-525nt)	P1	GCTTTAGGGATAACAGGGTAATTTAATTAACATCATCAATAATATACCGCACACTTTT
	P2	GTTTAAACGTCTGTTCCGCTGAGAGAAAACTCTAC
CMV cassette	P3	GTTTTCTCTCAGCGGAACAGACGTTTAAACTCCTCTTGCAGGTACGTG
	P4	CATGGAGCGGGTGGTGCTGCAGCTGCTGCCGCAGCTCCGCCAGCGCCTG
PAV3 genome (3273nt-5678nt)	P5	GCTGCGGCAGCAGCTGCAGCACCACCCGCTCCATGTTTC
	P6	CATCTTCCCATCAGCCCCCTCTCCTTAAGTGTCTGTTCCAGAAGCTGAG
PAV3 genome (33848nt-34094nt)	P7	CTCAGCTTCTGGAACAGACACTTAAGGAGAGGGGGCTGATGGGAAGATGG
	P8	AATTCTAGGGATAACAGGGTAATTTAATTAACATCATCAATAATATACCGCACACTTTT
CcdB-Cm cassette	P9	GACTCACTATAGGCTAGCGTTTAAACTAGCGACATCGATCACAAG
	P10	GAGGCTGATCAGCGGGTTTAAACTGATCTGTCGAATCGATCAC
EGFP gene	P11	GACTCACTATAGGCTAGCGTTTCGCCACCATGGTGAGCAAGG
	P12	GAGGCTGATCAGCGGGTTTACTTGTACAGCTCGTCCATGC
Firefly luciferase gene	P13	GACTCACTATAGGCTAGCGTTTGGTAAAGCCACCATGGAAGAC
	P14	GAGGCTGATCAGCGGGTTTCCCGACTCTAGAATTACACG
Lac Z gene	P15	GACTCACTATAGGCTAGCGTTTACGCCGCCACCATGTCGTTTACTTTG
	P16	GAGGCTGATCAGCGGGTTTATTTTTGACACCAGACCAACTG
	T7 pro	TTAATACGACTCACTATAGGG
	BGHpA rev	TAGAAGGCAGAGTCGAGG

In addition, a plasmid, pFPAV3, containing an E1-deleted PAdV-3 genome was generated by recombination between *Pst*I-linearized pPAV3 and the linear genomic DNA extracted from PAV219-infected VR1BL cells according to the SLiCE cloning method (Fig 1).

To establish a universal and zero background cloning method for foreign gene insertion, we further cloned the positive/negative double selection marker CcdB-Cm into the unique *Pme*Irestriction site of the intermediate genome plasmid pFPAV3 (Fig 1). Briefly, a 1786-bp fragment containing the CcdB-Cm expression cassette was amplified using primers (P9 and P10 in Table 1) and pAd-PL/Dust DNA as the template. As the 1786-bp fragment also contains 20 bp at each end that are homologous to the respective ends generated by *Pme*Idigestion of plasmid pFPAV3, the recombinant pFPAV3-CcdB-Cm plasmid was generated by the SLiCE cloning method using the 1786-bp fragment containing CcdB-Cm and *Pme*I-digested pFPAV3 (Fig 1).

#### Incorporation of a foreign gene into pFPAV3-CcdB-Cm based on SLiCE cloning and lethal CcdB screening

**Fig 2:**
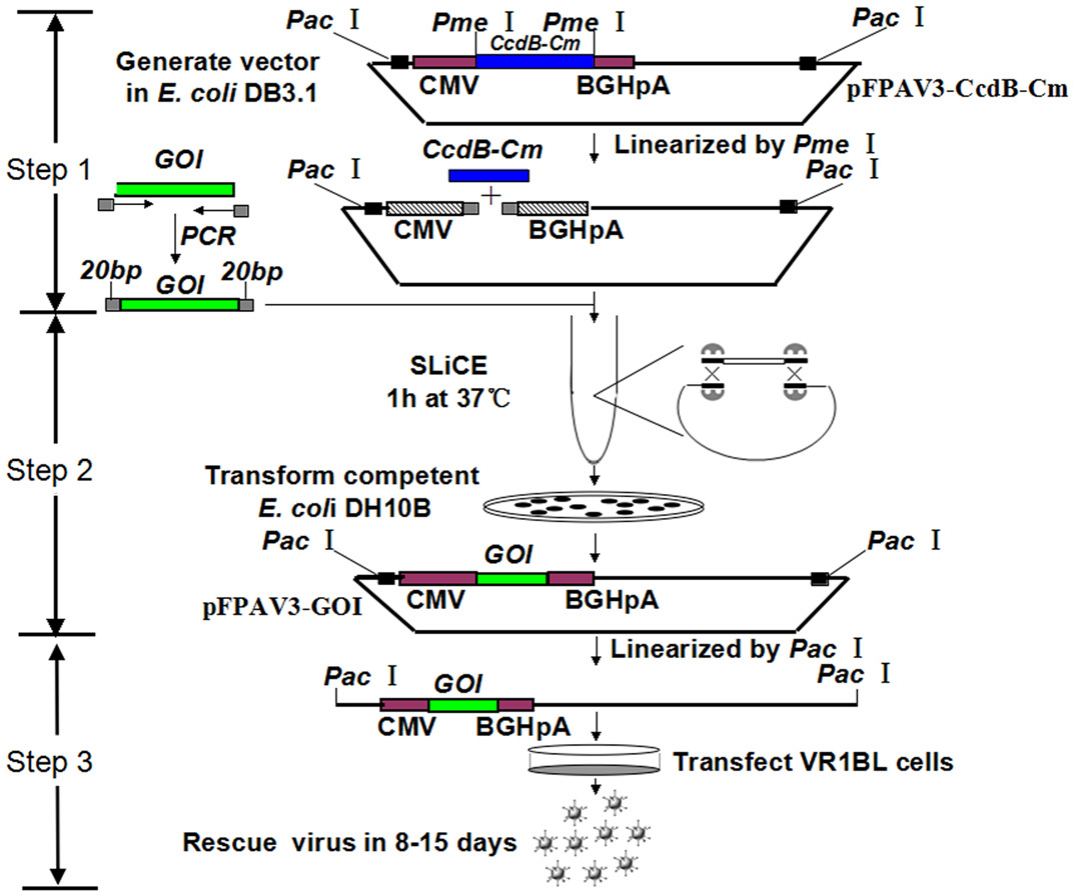
Schematic of recombinant porcine adenoviral vector construction and viral packaging. The recombinant PAdV-3 vector was constructed based on the SLiCE strategy by recombination between the *Pme*I-linearized genome vector pFPAV3-CcdB-Cm and a PCR fragment of a foreign gene containing 20-bp homologies. After a one-hour/one-tube *in vitro* reaction at 37°C, the SLiCE reaction mixture was subsequently transformed into DH10B competent cells and spread on plates containing kanamycin. Randomly selected clones were further confirmed by restriction enzyme analysis and sequencing. The positive recombinant PAdV-3 vectors were linearized by *Pac*I to liberate both inverted terminal repeats (ITRs) and then transfected into VR1BL cells for viral rescue.

To determine the foreign gene cloning efficiency based on SLiCE cloning and lethal CcdB screening, we constructed recombinant plasmids pFPAV3-EGFP, pFPAV3-Fluc and pFPAV3-LacZ by cloning as the inserts the reporter genes EGFP (plasmid pEGFP-N1, Clontech), firefly luciferase (plasmid pG5-Luciferase, Promega) and LacZ (plasmid pAAV-LacZ, Cell Biolabs) into pFPAV3-CcdB-Cm between the two *Pme*Isites instead of the CcdB-Cm cassette (Fig 2).

#### Identification the efficiency of the modified SLiCE method

The efficiency of the modified SLiCE method is determined by both cloning efficiency and cloning accuracy. The cloning efficiency are given as colonies amount post recombination with per nanogram of vector (vector: insert = 1: 10 molar ratio). The cloning accuracy is given as the percentage of positive clones among 48 randomly selected clones on kanamycin plates. The positive clones were determined by restriction enzyme digestion (*Hind* III or *EcoR* I) and sequencing with primers T7 pro and BGHpA rev in Table 1. In this study, we used the sequencing results to identify whether the foreign genes were exactly inserted into recombinant plasmids. Thus the positive sequencing results only represent the correct junctions post foreign genes insertion.

### Isolation of recombinant adenoviruses

Monolayers of VR1BL cells grown in a six-well dish were separately transfected with *Pac* I-linearized pFPAV3-EGFP, pFPAV3-Fluc and pFPAV3-LacZ DNA using Lipofectin Reagent (Invitrogen) according to the manufacturer’s recommendations. Cells showing cytopathic effects were collected, freeze-thawed and propagated for 3–4 passages. The titers of the corresponding rPAV3s were determined using the 50% tissue culture infective dose (TCID_50_) assay [21].

### Virus growth

The PAdV-3s growth properties were determined by infectious titer, also called the titer of infectious virions post PAV219 or individual recombinant viruses infection(at 0.1 MOI and infection 1×10^5^ VR1BL cells). At the indicated times post infection, the infected VR1BL cells were lysed by five rounds of freeze-thaw, and the titer of infectious virions was calculated by counting the average TCID_50_ of triple repeat according to Reed—Muench method [21].

### Virus transduction

To evaluate the expression of target genes, ST cells (1×10^5^ cells /well of a six-well plate) plated 1 day prior to infection were infected with 1 and 100 MOIs of the indicated recombinant PAdV-3s. At 24 h post-infection, the cells were analyzed. a) EGFP expression in PAV3-EGFP-infected cells was observed using fluorescent microscopy and analyzed by FACS (Beckman Coulter). b) Firefly luciferase signals were detected in pFPAV3-Fluc-infected cells lysates using the Luciferase Report assay system (Promega) in accordance with the manufacturer’s instructions. c) β-Galactosidase expression in PAV3-LacZ-infected cells was determined by X-gal staining, as described previously [22].

## Results

### Development of a porcine adenoviral vector system

To rapidly generate a recombinant porcine adenovirus, a novel vector system was constructed (Fig 1). The resulting plasmid was named pFPAV3-CcdB-Cm and had the following features. First, a gene expression cassette comprising a CMV promoter and BGHpA was placed in the E1 region, which is useful for expression of the transgene *in vitro* and *in vivo*. Second, the vector contains *Pme* I restriction enzyme sites between the CMV promoter and BGHpA for foreign gene insertion using the SLiCE cloning method *in vitro*. Third, the lethal CcdB gene and Cm gene expression cassettes were placed between the two *Pme* I restriction enzyme sites, allowing recombinants to be selected through lethal CcdB screening.

### Efficiency of foreign gene cloning into pFPAV3-CcdB-Cm

**Fig 3:**
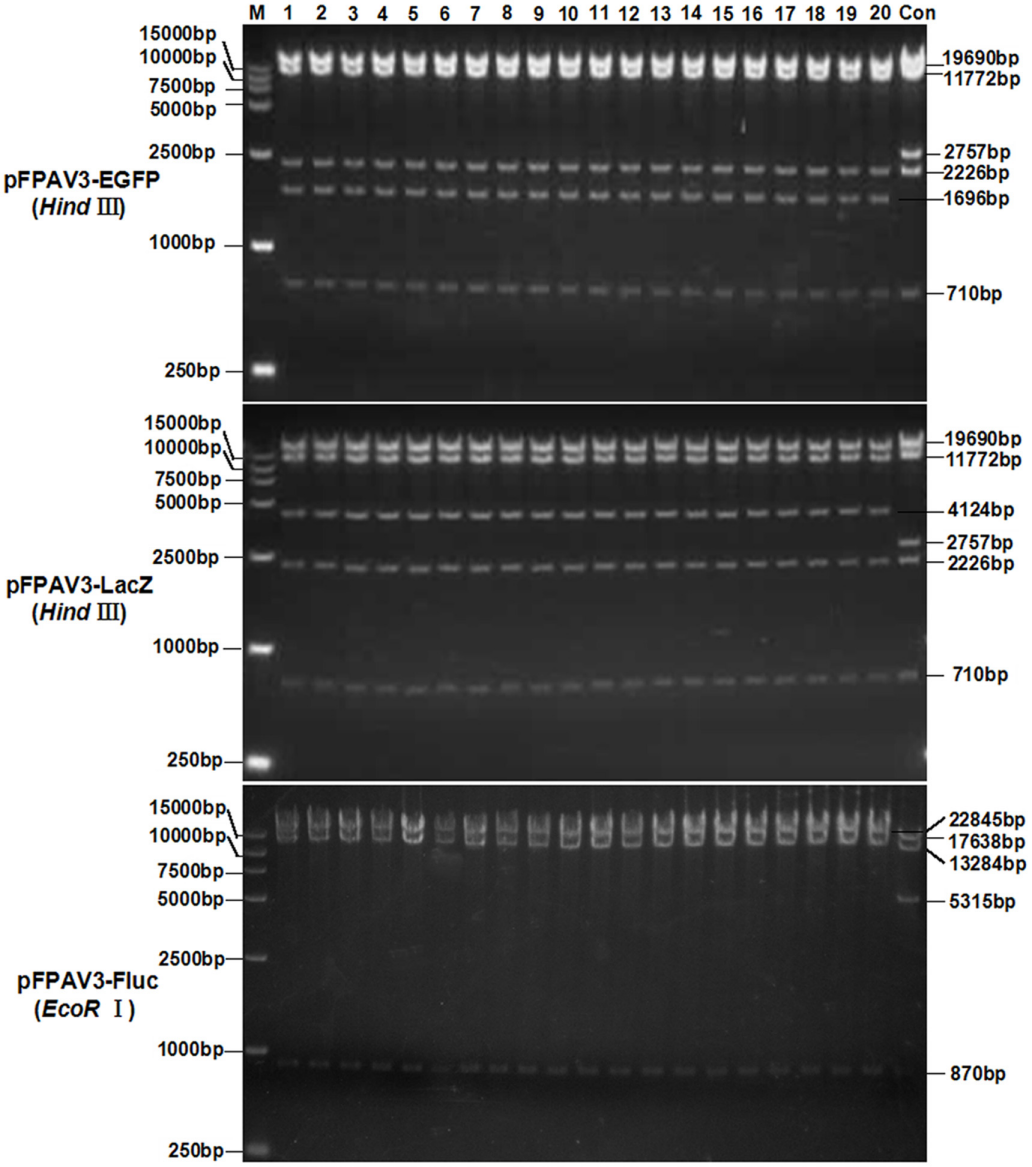
Restriction enzyme analysis of the recombinants drivered from the modified SLiCE cloning. After lethal CcdB gene screening on kanamycin plates, 48 randomly selected clones for the recombinant PAdV-3 vectors expressing each reporter gene were identified by *Hind* III digestion analysis. Lanes 1–20 of each group are the *Hind* III digestion of 20 clones among 48 selected clones. Lane Con is the *Hind* III digestion of the pFPAV3-CcdB-Cm parent vector which was used as the negative control for recombinant PAdV-3 vectors identification. Marker, DL15000.

To identify the cloning efficiency based on the SLiCE strategy, three reporter genes, EGFP, Fluc and LacZ, were individually inserted into the pFPAV3-CcdB-Cm vector to generate recombinant adenoviral vectors pFPAV3-EGFP, pFPAV3-Fluc and pFPAV3-LacZ, respectively (S1 Fig). After screening by lethal CcdB on 50 mg/L kanamycin plates, DNA from randomly selected bacterial colonies was isolated, digested with *Hind* III or *EcoR* I and analyzed by agarose gel electrophoresis. As expected (S2–S5 Figs And Fig 3), fragments of 19,690 bp, 11,772 bp, 2,757 bp, 2,226 bp and 170 bp were detected for the *Hind* III-digested plasmid pFPAV3-CcdB-Cm, and the 2,757-bp fragment in the *Hind* IIIrestriction enzyme map of pFPAV3-CcdB-Cm was replaced with a 971-bp, 2,649-bp or 4,124-bp fragment in pFPAV3-EGFP, pFPAV3-Fluc and pFPAV3-LacZ, respectively. As the *Hind* III restriction enzyme maps between plasmid pFPAV3-CcdB-Cm and pFPAV3-Fluc were similar and the *EcoR* I site in the CcdB/Cm gene of pFPAV3-CcdB-Cm was missing in FLuc insertion of pFPAV3-Fluc, we separately distinguished the recombinant pFPAV3-Fluc from parental plasmid pFPAV3-CcdB-Cm with *EcoR* I restriction enzyme digestion. As shown in Fig 3, fragments of 17,638 bp, 13,284 bp, 5,315 bp and 870 bp were detected for the *EcoR* I-digested plasmid pFPAV3-CcdB-Cm as predicted. The 5,135bp—*EcoR* I fragment in the restriction enzyme map of pFPAV3-CcdB-Cm was diminished in *EcoR* I-digested plasmid pFPAV3-Fluc. Instead, the 17,638 bp fragment in *EcoR* I-digested map of pFPAV3-CcdB-Cm was replaced by 22,845 bp (Fig 3). Moreover, the restriction enzyme analysis confirmed that all the randomly selected clones were positive for insertion of the reporter gene. The clone accuracy was further proven by DNA sequencing with the sequencing primer T7 pro and BGHpA rev (Table 1) of the plasmid pCDNA3.1(+) (data not shown). The efficiencies for EGFP (0.7 kb), Luc (1.7 kb) and LacZ (3.1 kb) cloning based on our modified SLiCE strategy (SLiCE cloning and lethal CcdB screening) was as high as 620±49.56, 490±26.88 and 370±50.99 clones/ng of plasmid, respectively. And the cloning accuracy were almost 100% positive after *Hind* III or *EcoR* I restriction enzyme analysis and the junctions sequencing (data not shown)(Table 2).

**Table 2:** Comparison of the cloning efficiencies for different insertion lengths.

Vector ^a^	Insert ^b^	Cloning efficiency ^c^	Cloning accuracy (%)^d^
Linear pFPAV3-CcdB-Cm Vector	EGFP (0.7 kb)	620±49.56	100
	Luc (1.7 kb) ^e^	490±26.88	100
	LacZ (3.1 kb)	370±50.99	100

^a^. The linear pFPAV3-CcdB-Cm vector was generated by *Pme* I digestion.

^b^. The EGFP, Luc and LacZ inserts flanked with 20-bp homologies were generated by PCR.

^c^. The cloning efficiencies of different insertion length are represented by colonies for per nanogram vector.

^d^. The cloning accuracy is represented by the percentage of positive colonies among the total identified clones after restriction enzyme analysis and sequencing.

^e^. Luc: Firefly luciferase gene.

### Analysis of recombinant PAV3-EGFP, PAV3-Fluc and PAV3-LacZ

**Fig 4:**
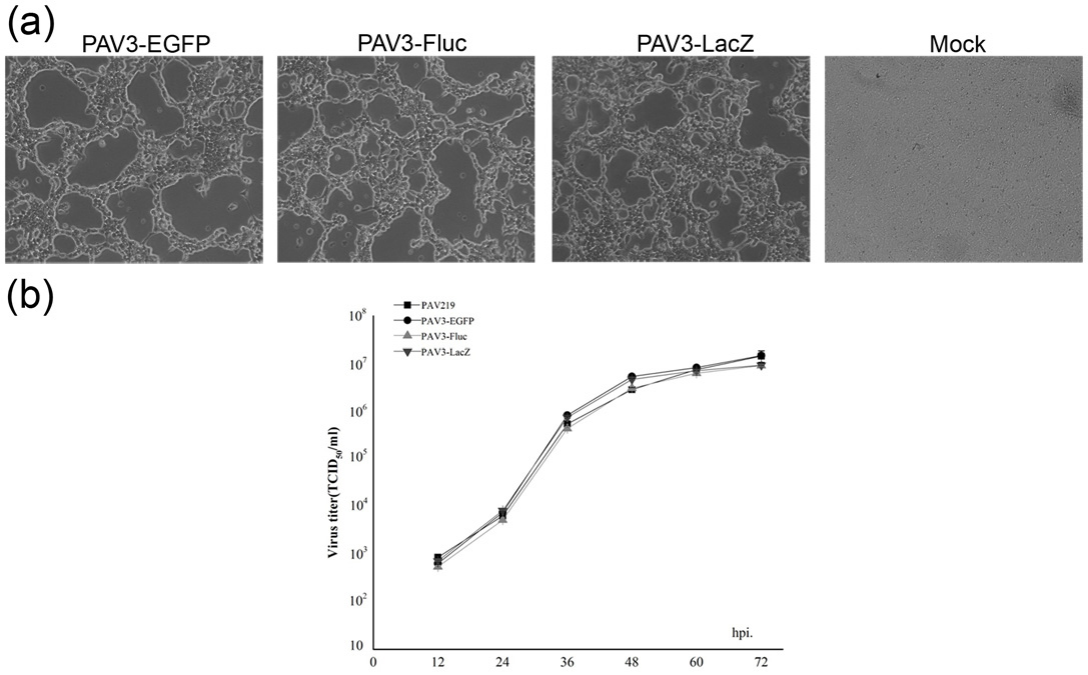
Generation of recombinant porcine adenoviruses PAV3-EGFP, PAV3-Fluc and PAV3-LacZ. (a) Cytopathic effects in VR1BL cells at 9 days after transfection with 5 μg pFPAV3-EGFP, pFPAV3-Fluc or pFPAV3-LacZ plasmid. (b) Growth kinetics of PAV219, PAV3-EGFP, PAV3-Fluc and PAV3-LacZ. Confluent monolayers of VR1BL cells were infected at an MOI of 0.1 with the PAV219 control or a recombinant PAdV-3 vector expressing the reporter gene. The infected VR1BL cells were harvested at the indicated times post-infection, and the amount of virus in cell lysate was determined according to TCID_50_. The viral titers are from the average of triplicate experiments.

**Fig 5:**
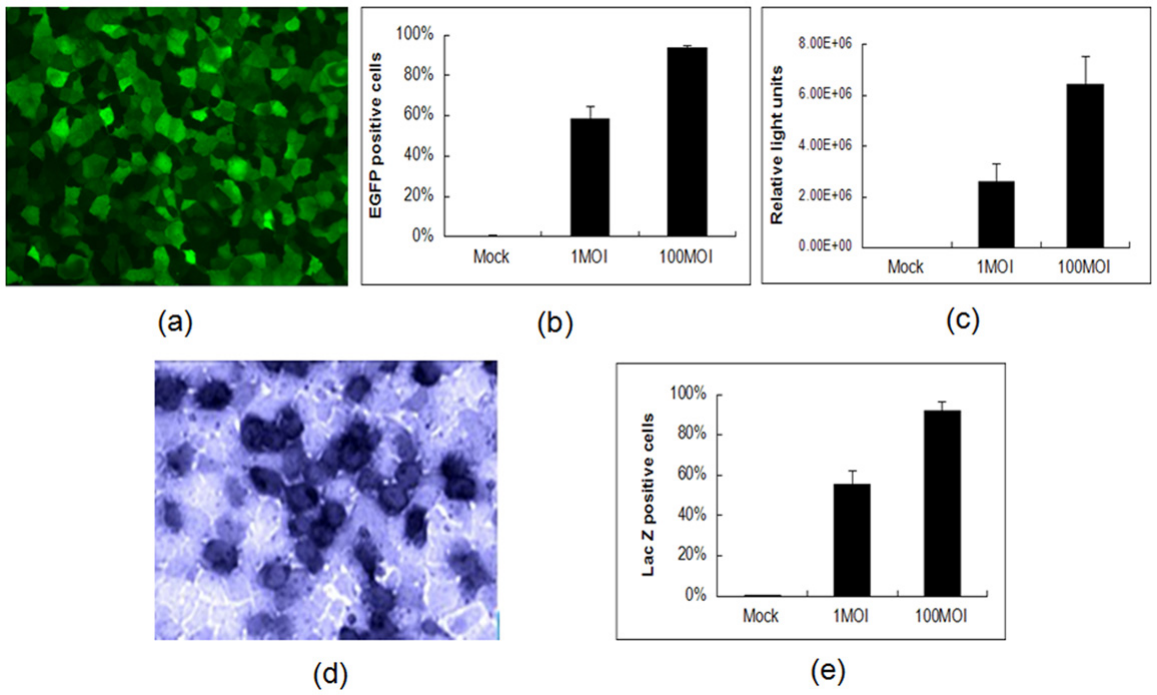
Reporter gene expression in ST cells at 24 h after viral transduction. (a) Expression of the EGFP gene was monitored by fluorescent microscopy. (b) The transduction efficiency in ST cells was analyzed by FACS. (c) Expression of the firefly luciferase gene was evaluated by a luciferase reporter assay in accordance with the manufacturer’s instructions. (d) Expression of the Lac Z gene in ST cells was evaluated by X-gal staining [22]. (e) The PAV3-Lac transduction efficiency in ST cells was evaluated by microscopy. The X-gal-staining cells were counted, and the percent transduced is the number of lac Z-positive cells relative to the total.

The plasmids pFPAV3-EGFP, pFPAV3-Fluc and pFPAV3-LacZ were individually transfected into VR1BL cells for the isolation of recombinant porcine adenoviruses. As shown Fig 4a, typical cytopathic effects including cell rounding, morphological irregularity, swelling/shrinking and detachment were observed at 9 days post-transfection. The infected cells were collected and freeze-thawed three times, and recombinant viruses PAV3-EGFP, PAV3-Fluc and PAV3-LacZ were proliferated in VR1BL cells. To determine growth properties or infectious titer of PAV219 or individual recombinant viruses, virus-infected VR1BL cells (at 0.1 MOI and infection 1×10^5^ cells) were collected at different times post-infection and analyzed by a TCID_50_ assay. As observed in Fig 4b, the recombinant PAdV-3s grew as efficiently as PAV219, and reporter gene expression was successfully detected in the virus- transduced ST cells (Fig 5).

## Discussion

Human adenovirus type 5 (HAdV-5)-based vectors have emerged during the last two decades as a promising gene delivery platform for a variety of therapeutic and vaccine purposes. However, HAdV-5 vector might induce associated acute toxicity as well as diminish the efficacy of therapeutic targeting following systemic administration in patient, due to the interactions between HAdV-5 capsid proteins and its receptors in the target cells including: (1) activation of macrophages, natural killer (NK) cells and classical complement pathway; (2) high transduction of immune cells in the liver and spleen; (3) induce inflammatory cytokines secretion[23–25]. In addition, the pre-existing immune of HAdV-5 further increases the histologic hepatic damage and limits the efficacy of gene therapy and vaccine development based on HAdV-5 vector [26–28]. To overcome these drawbacks, recombinant adenoviral vectors based on different genetic modifications of HAdV-5 capsids, different Helper-Dependent Adenoviral (HD-Ad) Vectors, less prevalent HAdV serotypes vectors (such as HAdV-11, HAdV-26 and HAdV-35) and nonhuman adenoviral vectors from different species (such as canine, bovine, porcine, murine, fowl, ovine and simian) were being evaluated [19, 29–36]. As one alternative non-human adenovirus, porcine adenovirus serotype 3 (PAdV-3) has many qualities which make it an ideal choice for use as a delivery vector, such as low grade pathogen, species specific infection following administration, knowledge of full length genome sequence, wide cell transduction in different species of including humans and other animals, circumvention of HAdV-5 vector-specific neutralizing antibody response [37–39]. Nonetheless, the isolation of recombinant PAdV-3 vectors by available methods [19, 34–36] is either time-consuming or expensive.

To date, many conventional systems for recombinant adenoviral vector construction have been established in accordance with *in vitro* cloning methods by digestion-ligation, Gateway or In-Fusion technology, and *in vivo* cloning methods by RecA, λ prophage Red, Cre/LoxP or Flp/Frt recombination in *E*. *Coli* [40–46]. Based on aforementioned coloning technology, four commercial systems, Adeno-X Expression SystemI(Clontech), Adeasy (Stratagene), ViraPower Adenoviral Expression SystemI(Invitrogen) and Adeno-X Expression System 3 (Clontech), have been widely applied all over the world. For rare restriction enzymes, ligation of Adeno-X Expression System I and RecA recombination in the Adeasy system, the gene cloning efficiency is typically variable (from 20% to 90%) due to the contamination of non-cutting adenoviral plasmid, non-cutting shuttle plasmid, and/or difficulty in large-fragment ligation. Although the gene cloning efficiency and accuracy for ViraPower Adenoviral Expression System and Adeno-X Adenoviral System 3 is nearly 100%, these two systems are highly dependent on the use of expensive restriction enzyme-mediated Gateway and In-Fusion cloning (S6 Fig and S1 Table).

The *in vivo* λ prophage Red system for recombinant adenoviral vector construction reported by Samuel Campos and Michael Barryis depends on the recombination in strain BW25113 harbors pKD46, a temperature-sensitive plasmid encoding the Red genes under control of the pBAD L-arabinose inducible promoter, and FLP-mediated removal of the FRT-flanked ZeoR cassettes in DH5α strain harboring pCP20, a chloramphenicol-resistant temperature sensitive plasmid for the expression of FLP recombinase[44]. Different from the conventional *in vivo* cloning based on λ prophage Red recombination, we developed a novel, efficient and zero-background cloning method for the generation of recombinant PAdV-3 vectors based on SLiCE cloning and lethal CcdB screening in this study. The SLiCE cloning is one type of *in vitro* cloning methods based on λ prophage Red recombination with 20–40 bp homology. As illustrated in Fig 2, the novel strategy developed involves three major steps. First, the gene of interest containing 20–40 bp homologies is generated by PCR. Second, the gene of interest flanked with 20–40 bp homologies is cloned into the backbone vector based on the SLiCE cloning method and lethal CcdB gene screening. Multiple restriction digestion analysis is performed for further identification. Third, the recombinant porcine adenoviral plasmid is cleaved with *Pac*Ito expose its inverted terminal repeats and then transfected into a packaging cell line (VR1BL cells). The SLiCE cloning method, the positive selection by the lethal CcdB gene and the ability to bypass further identification steps make the construction of porcine adenoviral vectors using this approach simple and efficient.

Comparing with the reported methods for recombinant adenoviral vector construction, our modified SLiCE system has several advantages. First, a shuttle plasmid and restriction enzyme-mediated ligation is not needed. The gene of interest with flanking 20–40 bp homologies is generated by a single PCR amplification and is directly cloned into a linearized adenoviral plasmid in a one-hour/one-tube *in vitro* reaction followed by standard transformation of host bacteria. Second, the cloning efficiency can reach as high as 620±49.56 clones/ng of plasmid, and the cloning accuracy based on the SLiCE cloning and lethal CcdB screening strategy was almost 100% by restriction enzyme analysis and the insertion junctions sequencing (Not sure whether all the recombinant clones on plate were 100% positive). Third, by avoiding the steps to prior the treatment of end sequences by enzymes, SLiCE cloning is very cost efficient. Finally, the virus growth and transduction efficiency of the recombinant PAdV-3 vectors isolated using the SLiCE system appeared to be comparable to a recombinant PAdV-3 isolated using a conventional system. The detailed comparison of our modified SLiCE system with current commercial systems is provided in S6 Fig and S1 Table.

To our knowledge, the method described herein is the first successful example of the application of the SLiCE cloning method to the construction of recombinant adenoviral vectors. The CcdB-Cm marker in pFPAV3-CcdB-Cm cloning plasmid was used for double screening of the positive clones for the recombinant adenoviral plasmid, by which non-recombinant or partially recombinant clones are eliminated [47]. The Cm marker could be further used to detect the contamination of non-recombinant pFPAV3-CcdB-Cm vectors, though the Cm screening step in our work was replaced by the step of restriction enzyme analysis.

In summary, our porcine adenovirus system based on the SLiCE cloning method and lethal CcdB gene screening is an easy, efficient and inexpensive cloning method that allows the generation of recombinant porcine adenoviral vectors in a seamless and precise fashion. Although we evaluated and established this versatile cloning method for modification the E1 region of PAdV-3 vectors in this study, this method can also be utilized to modify the other regions such as fiber, hexon or pIX within the PAdV-3 genome, as well as the adenoviral vectors based on human and other non-human adenovirus.

## Supporting Information

S1 Fig Schematic of recombinant pocine adenoviral vectors contianing different transgenes. The genes for EGFP (0.7kb), firefly luciferase (1.7kb) and lac Z (3.1kb) flanked with 20-bp homologies were cloned into the pFPAV3-CcdB-Cm vector using the SLiCE strategy
(TIF)

S2 Fig The map of the parent plasmid pFPAV3-CcdB-Cm. The *Hind* III and *EcoR*Isites were shown in the maps of the parental plasmid pFPAV3-CcdB-Cm. The *Hind* III sites and *Hind* III cleavage fragment for differentiation three recombinant constructions from the parental plasmid pFPAV3-CcdB-Cm were labeled in red. In addition, the unique *EcoR*Isite within the CcdB-Cm gene of the parental plasmid pFPAV3-CcdB-Cm was also labeled in red, which was only used for differentiation the plasmid pFPAV3-Fluc from parent plasmid pFPAV3-CcdB-Cm as this *EcoR*Isite was missing in plasmid pFPAV3-Fluc.
(TIF)

S3 Fig The map of the result plasmid pFPAV3-EGFP. The *Hind* III and *EcoR*Isites were shown in the maps of the plasmid pFPAV3-EGFP. The *Hind* III sites and *Hind* III cleavage fragment containing the EGFP gene were labeled in red.
(TIF)

S4 Fig The map of the result plasmid pFPAV3-Fluc. The *Hind* III and *EcoR*Isites were shown in the maps of the plasmid pFPAV3-EGFP. The *Hind* III sites and *Hind* III cleavage fragment containing the Fluc gene were labeled in red.
(TIF)

S5 Fig The map of the result plasmid pFPAV3-LacZ. The *Hind* III and *EcoR*Isites were shown in the maps of the plasmid pFPAV3-LacZ. The *Hind* III sites and *Hind* III cleavage fragment containing the LacZ gene were labeled in red.
(TIF)

S6 Fig Schematic representation of the construction of recombinant adenoviral vectors. (a) Foreign gene cloning based on Adeno-X Expression SystemI. The foreign gene was cloned into the MCS between I-*Ceu*I and PI-*Sce*I sites of the shuttle plasmid pShuttle. Then, the shuttle vector and the genome vector were both double digested with I-*Ceu*Iand PI-*Sce*I for *in vitro* ligation. To avoid the contamination of un-cut plasmid, the ligation products were pre-digested by *Swa*I before transformation. (b) Foreign gene cloning based on the Adeasy system. The foreign gene was cloned into the MCS of the shuttle plasmid pAd-Shuttle. Then, the *Pme*I-linearized pAd-Shuttle-GOI plasmid was transformed into BJ5183 competent cells pre-transformed with genome plasmid pAdeasy-1. After recombination, the DNA of kanamycin-resistant clones was isolated from BJ5183 cells and re-transformed into other high-copy strains to produce a large amount of viral vector. (c) Foreign gene cloning based on the Gateway system. The foreign gene was cloned into the MCS of the shuttle vector p-Entry. Then, the GOI was transferred from the shuttle plasmid p-Entry-GOI into the adenoviral vector pAd-CMV-Dust by LR clonase-mediated recombination. (d) Foreign gene cloning based on Adeno-X Expression System 3. The foreign gene flanking with 20-bp homologies was cloned into the adenoviral vector by In-Fusion enzyme-mediated recombination. (e) Foreign gene cloning based on our modified SLiCE system. The foreign gene flanking with 20-bp homologies was cloned into the adenoviral vector using the crude cell extracts of *Escherichia coli* strain PPY after induction with L-arabinose.
(TIF)

S1 Table Comparison of four methods for recombinant adenoviral vector construction. (DOC)
