# Supplementary figures and images for: A Novel and Simple Method for Rapid Generation of Recombinant Porcine Adenoviral Vectors for Transgene Expression
Source: PLoS One. 2015 May 26;10(5):e0127958. doi: 10.1371/journal.pone.0127958 (PMC4444375; doi:10.1371/journal.pone.0127958)

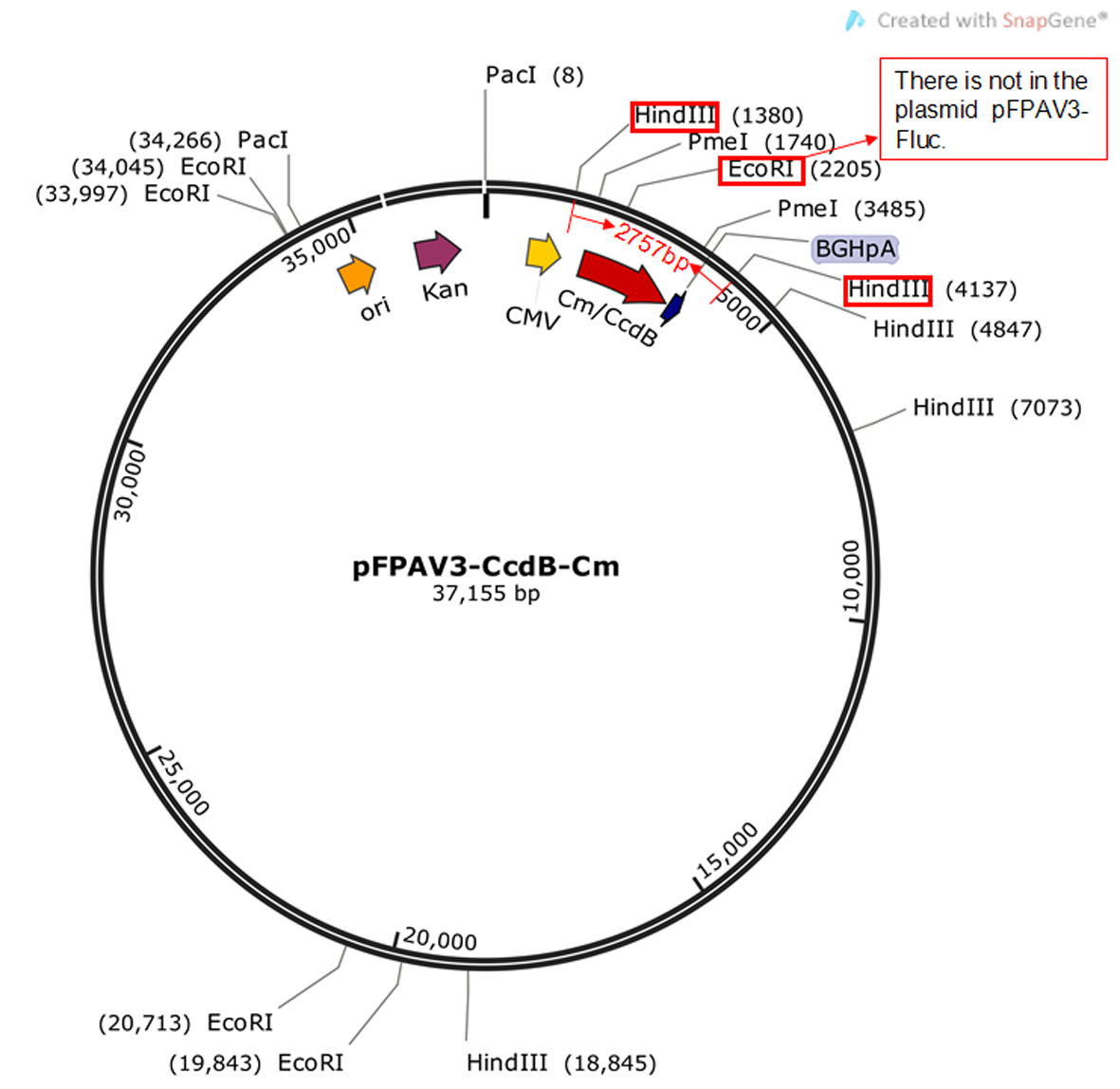

Supplement: S2 Fig — The Hind III and EcoRIsites were shown in the maps of the parental plasmid pFPAV3-CcdB-Cm. The Hind III sites and Hind III cleavage fragment for differentiation three recombinant constructions from the parental plasmid pFPAV3-CcdB-Cm were labeled in red. In addition, the unique EcoRIsite within the CcdB-Cm gene of the parental plasmid pFPAV3-CcdB-Cm was also labeled in red, which was only used for differentiation the plasmid pFPAV3-Fluc from parent plasmid pFPAV3-CcdB-Cm as this EcoRIsite was missing in plasmid pFPAV3-Fluc. (TIF) [file pone.0127958.s002.tif]

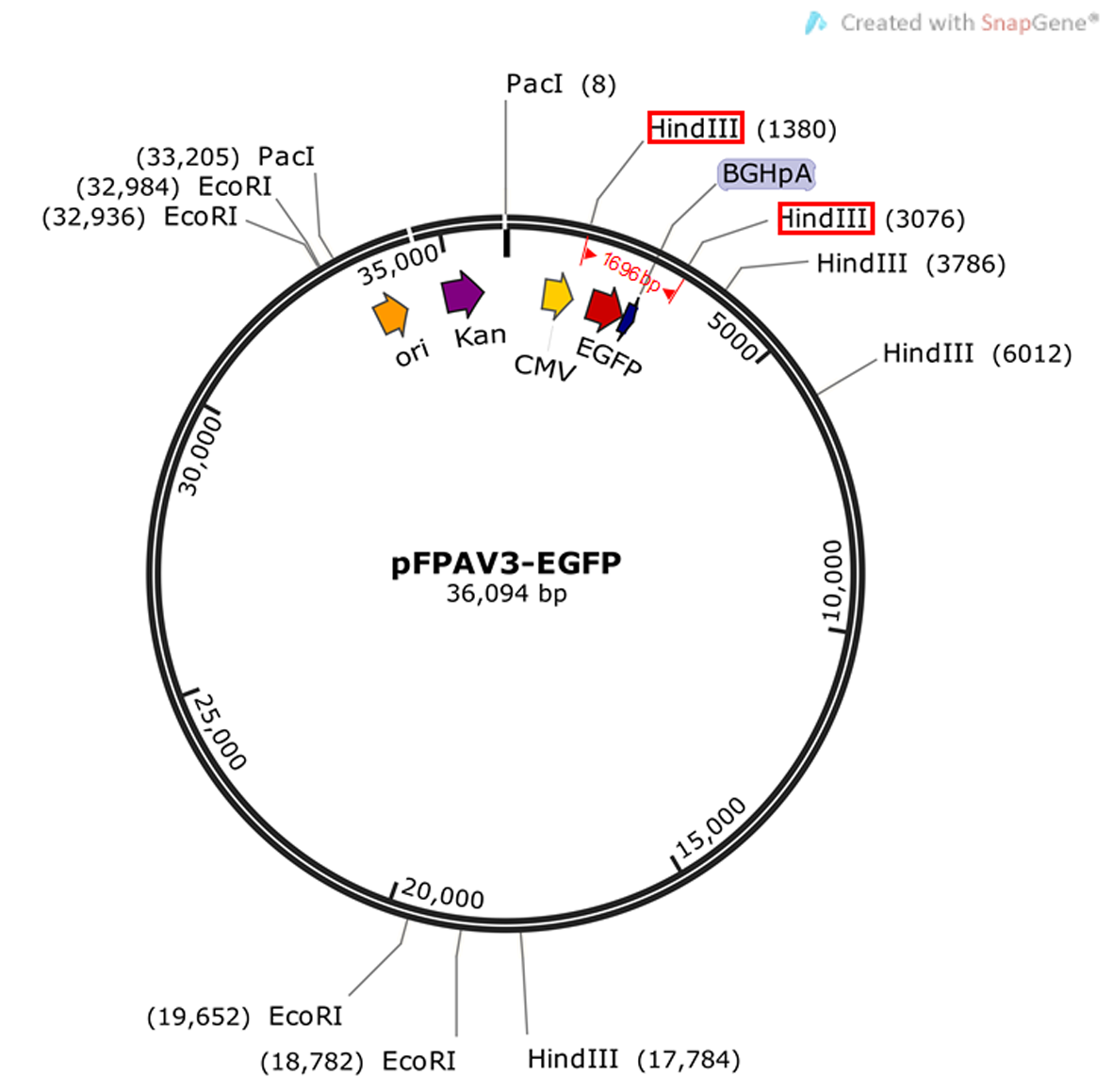

Supplement: S3 Fig — The Hind III and EcoRIsites were shown in the maps of the plasmid pFPAV3-EGFP. The Hind III sites and Hind III cleavage fragment containing the EGFP gene were labeled in red. (TIF) [file pone.0127958.s003.tif]

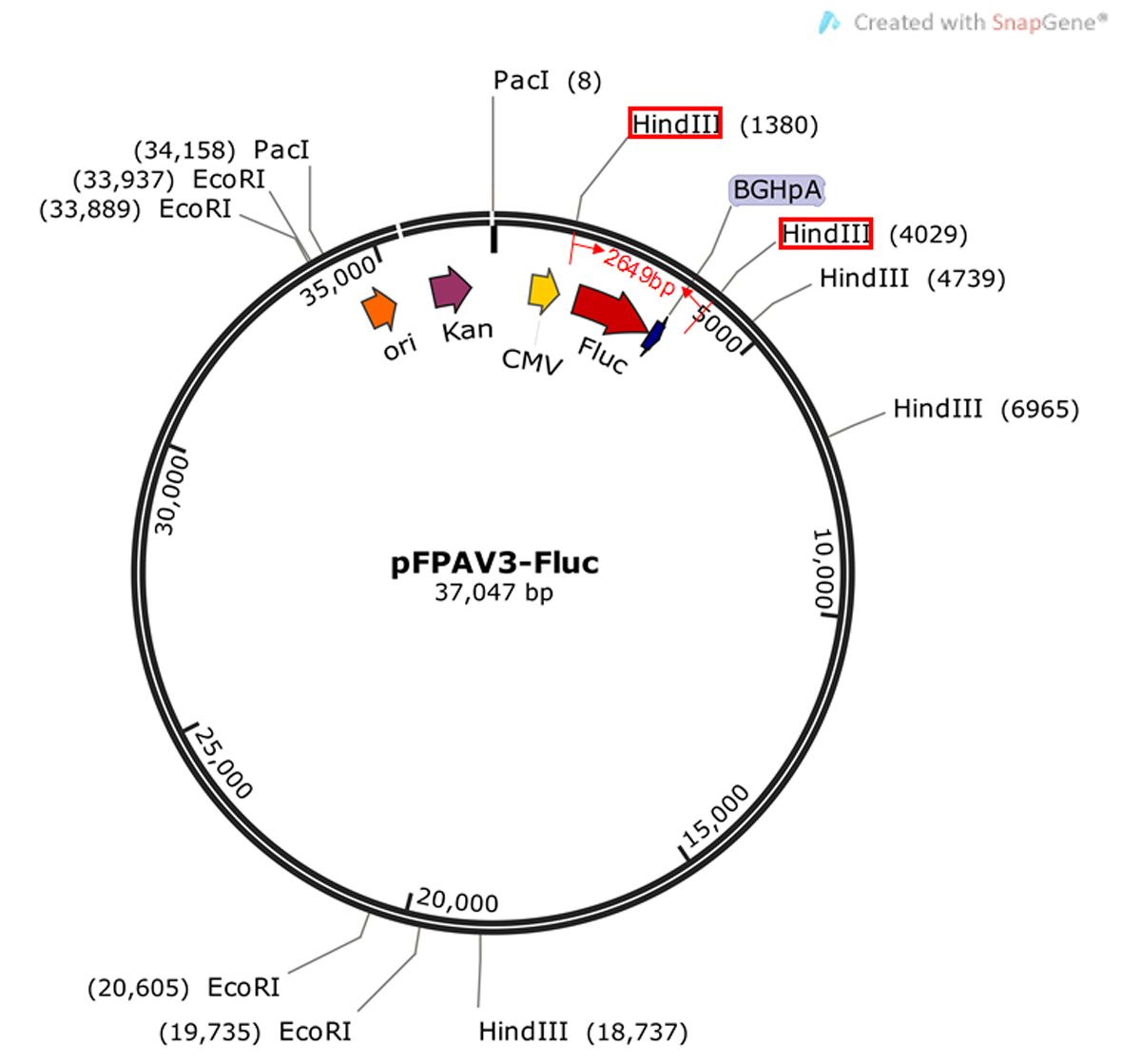

Supplement: S4 Fig — The Hind III and EcoRIsites were shown in the maps of the plasmid pFPAV3-EGFP. The Hind III sites and Hind III cleavage fragment containing the Fluc gene were labeled in red. (TIF) [file pone.0127958.s004.tif]

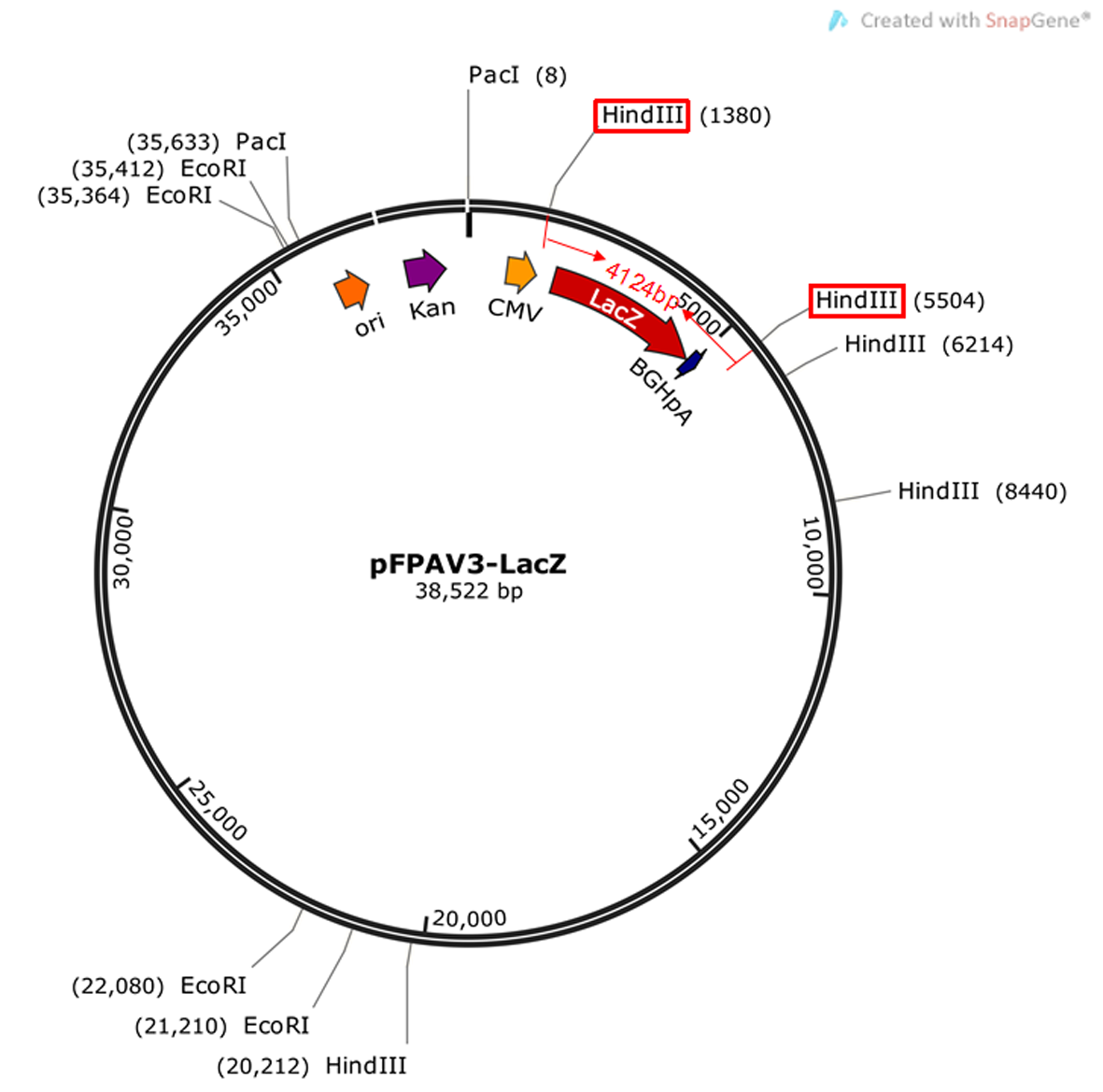

Supplement: S5 Fig — The Hind III and EcoRIsites were shown in the maps of the plasmid pFPAV3-LacZ. The Hind III sites and Hind III cleavage fragment containing the LacZ gene were labeled in red. (TIF) [file pone.0127958.s005.tif]

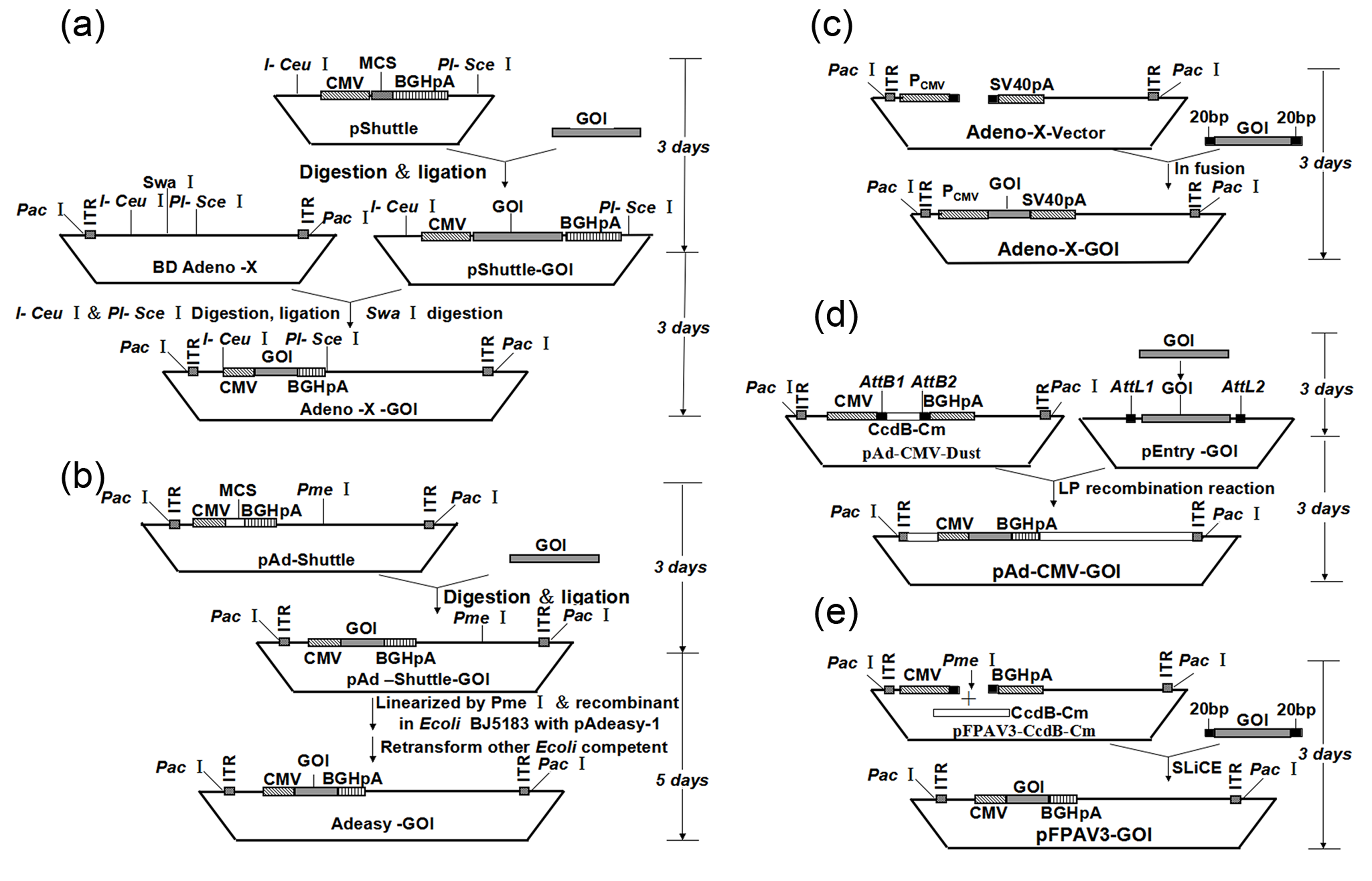

Supplement: S6 Fig — (a) Foreign gene cloning based on Adeno-X Expression SystemI. The foreign gene was cloned into the MCS between I-CeuI and PI-SceI sites of the shuttle plasmid pShuttle. Then, the shuttle vector and the genome vector were both double digested with I-CeuIand PI-SceI for in vitro ligation. To avoid the contamination of un-cut plasmid, the ligation products were pre-digested by SwaI before transformation. (b) Foreign gene cloning based on the Adeasy system. The foreign gene was cloned into the MCS of the shuttle plasmid pAd-Shuttle. Then, the PmeI-linearized pAd-Shuttle-GOI plasmid was transformed into BJ5183 competent cells pre-transformed with genome plasmid pAdeasy-1. After recombination, the DNA of kanamycin-resistant clones was isolated from BJ5183 cells and re-transformed into other high-copy strains to produce a large amount of viral vector. (c) Foreign gene cloning based on the Gateway system. The foreign gene was cloned into the MCS of the shuttle vector p-Entry. Then, the GOI was transferred from the shuttle plasmid p-Entry-GOI into the adenoviral vector pAd-CMV-Dust by LR clonase-mediated recombination. (d) Foreign gene cloning based on Adeno-X Expression System 3. The foreign gene flanking with 20-bp homologies was cloned into the adenoviral vector by In-Fusion enzyme-mediated recombination. (e) Foreign gene cloning based on our modified SLiCE system. The foreign gene flanking with 20-bp homologies was cloned into the adenoviral vector using the crude cell extracts of Escherichia coli strain PPY after induction with L-arabinose. (TIF) [file pone.0127958.s006.tif]
