# Supplementary material for: A Novel and Simple Method for Rapid Generation of Recombinant Porcine Adenoviral Vectors for Transgene Expression
Source: PLoS One. 2015 May 26;10(5):e0127958. doi: 10.1371/journal.pone.0127958 (PMC4444375; doi:10.1371/journal.pone.0127958)
Supplement: S1 Table — (DOC) [file pone.0127958.s007.doc]

**S1 Table Comparison of four methods for recombinant adenoviral vector construction**

|  | Adeno-X™ Expression  System | Adeasy^TM^  system | ViraPower^TM^ Expression System | Adeno-X^TM^  Expression System 3 | method  in the present study |
| --- | --- | --- | --- | --- | --- |
| Mechanism | Ligation in vitro | RecA-dependent  recombination in *E. coli* | In vitro site-specific recombination | In-Fusion | SLiCE *in vitro* |
| Screening | Antibiotic screening  and *Swa* I digestion | antibiotic screening | CcdB gene and  antibiotic screening | No | CcdB gene and  antibiotic screening |
| Sub cloning | yes | yes | yes | no | no |
| Cloning efficiency | Moderate (variable) | Moderate (60%) | High (>99%) | High (>99%) | High (>99%) |
| Time | 6 days | 8 days | 6 days | 3 days | 3 days |
| Cost | high | low | high | high | low |
